# Poly-Ub-Substrate-Degradative Activity of 26S Proteasome Is Not Impaired in the Aging Rat Brain

**DOI:** 10.1371/journal.pone.0064042

**Published:** 2013-05-07

**Authors:** Carolin Giannini, Alexander Kloß, Sabrina Gohlke, Michele Mishto, Thomas P. Nicholson, Paul W. Sheppard, Peter-Michael Kloetzel, Burkhardt Dahlmann

**Affiliations:** 1 Institut für Biochemie, Charité-Universitätsmedizin-Berlin, Berlin, Germany; 2 Enzo Life Sciences (UK) Ltd, Palatine House, Matford Court, Exeter, United Kingdom; Universidad de Sevilla, Spain

## Abstract

Proteostasis is critical for the maintenance of life. In neuronal cells an imbalance between protein synthesis and degradation is thought to be involved in the pathogenesis of neurodegenerative diseases during aging. Partly, this seems to be due to a decrease in the activity of the ubiquitin-proteasome system, wherein the 20S/26S proteasome complexes catalyse the proteolytic step. We have characterised 20S and 26S proteasomes from cerebrum, cerebellum and hippocampus of 3 weeks old (young) and 24 month old (aged) rats. Our data reveal that the absolute amount of the proteasome is not dfferent between both age groups. Within the majority of standard proteasomes in brain the minute amounts of immuno-subunits are slightly increased in aged rat brain. While this goes along with a decrease in the activities of 20S and 26S proteasomes to hydrolyse synthetic fluorogenic tripeptide substrates from young to aged rats, the capacity of 26S proteasomes for degradation of poly-Ub-model substrates and its activation by poly-Ub-substrates is not impaired or even slightly increased in brain of aged rats. We conclude that these alterations in proteasome properties are important for maintaining proteostasis in the brain during an uncomplicated aging process.

## Introduction

Limited as well as complete proteolysis of proteins are important mechanisms for regulation of biological processes. A major part of intracellular proteolysis is catalysed by the ubiquitin-proteasome system (UPS), which thus is involved in many vitally important cellular functions. Impairment of the UPS due to the ontogenetic development or on account of pathological alterations inevitably entails consequences for the integrity or even viability of a cell. Similarly, the process of aging goes along with changes in protein metabolism and with a slowed down turnover of intracellular proteins leading to an accumulation of dysfunctional proteins [1].

Within the UPS peptide bond hydrolysis is catalysed by the 20S proteasome moiety (20S). The 20S proteasome is a cylinder-shaped complex that is composed of four stacked rings, each consisting of seven protein subunits. Each of the two inner rings consists of β-subunits (β1–β7), three of which (β1, β2, β5) harbour the proteolytically active sites catalysing a caspase-like, trypsin-like and chymotrypsin-like activity, respectively. Functions of the α-subunits (α1–α7) composing the two outer rings are, e.g. gating a central pore enabling the entry of substrates into the inner proteolysis hole and binding of substrate proteins and/or regulator complexes like the 19S regulator (19S), PA28 and PA200, respectively. Binding of these regulator complexes leads to formation of multiple forms of proteasomes like 26S proteasomes, i.e. 19S-20S-19S, like PA28/20S/PA28 complexes and hybrid proteasomes (PA28/20S/19S) [2]. The activity of the proteasome has been investigated in several cells and tissues during aging. When measured in aged as compared to young rats reduced chymotrypsin-like activity of the proteasome was found in tissues like heart, liver, kidney, lung, spinal cord, but also in brain cortex and hippocampus. No decrease in proteasomal activity was found in the cerebellum and brain stem [3].

With few exceptions, these data were confirmed by other investigators [4]. However, the age-dependent effects on proteasome activity from different brain regions differed with regard to the extent of decline, the type of proteasome activity measured and species investigated [5], [6]. Several possible reasons for the age-dependent decrease in proteasome activity of neuronal tissue were presented and discussed, e.g. reduction in transcription of proteasome-subunit genes, chemical modification of proteasome subunits and/or inhibition by lipid peroxidation products due to unhampered oxidative stress [4]. Further age-related alterations are the exchange of standard catalytic β-subunits (β1, β2, β5) by interferon-γ-inducible, so-called immunosubunits (β1i, β2i, β5i) and/or reduction in the content of PA28 and 19S regulator [7], [8]. Since the specific activities of uncapped 20S proteasomes and of proteasomes associated with various regulators are considerably different [2], change of their ratio could explain the generally observed age-dependent decrease of proteasome activity.

So far however, a detailed functional analysis of age dependent changes in poly-Ub-substrate degradation capacity of 26S proteasomes is still missing. Here we separated 20S and 26S proteasomes from three different brain regions of young and aged rats, determined their amount and specific activities and characterized their subunit composition. While the hydrolytic activities of proteasomes, measured by short peptide-based fluorogenic assays, decreased during aging, the capacity for degradation of ubiquitinated proteins and susceptibility for activation by poly-Ub-substrates was conserved.

## Materials and Methods

### Animals

The study protocol was reviewed and approved by the Landesamt für Gesundheit und Soziales, Berlin, Germany (Permit Number: T0303/06).

Cerebrum, cerebellum and hippocampus from 3 week old and 24 month old male male Sprague Dawley rats were purchased from Zivic Laboratories Inc. (New Castle, PA, USA) and stored at –80°C until analysis. The protocols for animal breeding and sacrifice were approved in 1993 report of the American Vetenary Medical Association (AVMA) a member of the Association for Assessment and Accreditation of Laboratory Animal Care International (AAALAC).

### Antibodies

If not stated otherwise antibodies used were as follows: anti-proteasome subunit α1 (IB5, Cappel, Belgium); anti-proteasome subunit β1 (K43, laboratory stock); anti-proteasome subunit β5 (ab3330, abcam, U.K.); anti-proteasome subunit β1i (ab3328, abcam, U.K.); anti-proteasome subunit β5i (ab3329, abcam, U.K.); anti-UbcH5 (UBE2D1) and anti-GST (Sigma Aldrich, Germany); anti-USP14 (Bethyl Laboratories, U.S.A.); rabbit antiserum against 20S proteasome purified from rat skeletal muscle (serum 37) and rat liver (serum 87) (laboratory stock); peroxidase-labelled goat anti-rabbit and anti-mouse antibodies (Dianova, Germany).

### Purification of 20S and 26S proteasomes and separation of 20S proteasome subtypes

The fluorogenic peptide substrates succinyl-Leu-Leu-Val-Tyr-7-amido-4-methylcoumarin (Suc-LLVY-MCA), benzoyl-Val-Gly-Arg-MCA (Bz-VGR-MCA), and carbobenzoxy-Leu-Leu-Glu-MCA (Z-LLE-MCA) were purchased from Bachem AG (Switzerland) and used for measurement of the chymotrypsin-, trypsin-, and caspase-like activities, respectively, as described elsewhere [9]. Purification of 20S and 26S proteasomes to apparent homogeneity was performed as described elsewhere [10] with an additional purification step by subjecting the enzymes to a final glycerol gradient centrifugation as stated below.

Separation of 20S proteasomes into subtypes was performed by high resolution anion exchange chromatography on a Mini Q column using a SMART chromatography system as described elsewhere [9].

### Preparation of brain tissue extracts and glycerol gradient centrifugation

Cerebrum, cerebellum and hippocampus, respectively, were weighed and then suspended in a 2.5-fold volume (v/w) of 10 mM Tris/HCl, 1.1 mM MgCl_2_, 10 mM NaCl, 0.1 mM EDTA, 1 mM NaN_3_, 1 mM DTT, 2 mM ATP, 10% (v/v) glycerol, pH 7.0 (TSDG buffer) and homogenized in a Potter Elvehjem homogenizer at 4°C. The homogenates were centrifuged at 15000 *g* for 60 min and supernatants were used as tissue extracts. For further separation of 20S and 26S proteasomes the tissue extracts were loaded on a 19 ml linear gradient of glycerol (10–40% (v/v)) in TSDG buffer. Centrifugation was performed using a SW28 rotor in an ultracentrifuge (Beckman) at 25000 rpm for 24 hours at 4°C. The gradients were fractionated into fractions of 0.5 ml volume, which were then tested for proteolytic activity using Suc-LLVY-MCA as substrate. Fractions comprising the different peaks of proteolytic activity were pooled as shown in the result section. Preparation of tissue extracts for detection of poly-Ub-protein-conjugates was performed in TSDG buffer containing 0.1% (v/v) NP40, 10 mM N-ethylmaleimide and 100 µM MG132.

### Quantitative determination of proteasomes

Quantification of proteasome content was performed by rocket-immunoelectrophoresis [11]. Proteasome containing samples were applied to 1% (w/v) agarose gels containing 0.5% (v/v) antiserum against native rat 20S proteasome (mixture of rabbit serum 37 and 87) were run at 50V for 16 h in a horizontal electrophoresis chamber. Gels were then incubated with 7.5% (v/v) acetic acid, dried at room temperature and stained with Coomassie brilliant blue. The height of precipitates (rockets) was measured and the concentration of proteasome calculated by use of standard curves established with purified 26S and 20S proteasomes. The regression coefficient of the standard curves was r^2^ = 0.9898 for 20S proteasomes and r^2^ = 0.9875 for 26S proteasomes. The linear detection range for 20S and 26S proteasome was 50–1000 ng/ml.

### Determination of poly-Ub-substrate degradation by 26S proteasomes

The linearly tetramerized peptide (amino acids 950–958) of the mucin glycoprotein MUC1 fused N-terminally to ubiquitin, which was conjugated to tetra-ubiquitin at lysine-48, designated Ub_5_Muc_4_ substrate, was generously provided by Dr. Bech-Otschir and its degradation by 26S proteasome analysed as described [12]. 600 nM Ub_5_-Muc_4_ was dissolved in 50 mM Tris/HCl, pH 7.6, containing 10 mM KCl, 0,5 mM DTT, 5 mM MgCl, 2 mM ATP and incubated with 70 nM 26S proteasome at 37°C. Aliquots were taken at defined time points and the reaction stopped by freezing. The aliquots were subjected to SDS-PAGE and remaining substrate detected by immunoblotting using an antibody to Muc_950–958_. The proteasome α1-subunit was monitored by immunoblotting with antibody IB5 as loading control. The poly-ubiquitinated, GST-tagged UbcH5 was prepared as described elsewhere [13]. The degradation of this substrate was traced by incubation of 800 nM of substrate protein (calculated on the basis of a tetra-ubiquitinated substrate protein) with 70 nM of 26S proteasome under the same conditions as described above. Remaining poly-Ub-GST-UbcH5 was detected by immunoblotting with anti-UbcH5. Proteasome was monitored by use of proteasome antiserum 37. Binding of secondary antibodies was visualized by enhanced chemiluminescence technique. Quantification of the immunreactive protein bands was performed by densitometric analysis by means of the NIH Image J programme [14].

### Electrophoretic techniques and immunoblotting

Polyacrylamide gel electrophoresis under non-denaturing conditions and proteasome activity detection by substrate overlay technique was performed as described [10]. Quantification of the activity signal was performed by means of the ImageJ software. 2D-PAGE was performed in a non-equilibrium-pH-gradient system as described elsewhere [15]. Western semidry blotting of one-dimensional SDS polyacrylamide gels was performed using PVDF membrane. Primary antibodies bound to the membrane were detected by enhanced chemiluminescence (GE Healthcare) using peroxidase-labelled goat anti-rabbit or anti-mouse antibodies.

### Calculation and statistics

Reported values are means ± SEM. All statistical analysis was performed using SIGMASTAT 3.0. Observed differences in values between young and aged animals were analyzed for statistical significance by using the student's t test. Mann-Whitney Rank-Sum test was performed in case normality or equal variance failed.

## Results

### Total proteasome content in the brain areas of young and aged rats

Comparative investigations of proteasomes from the different brain regions were performed with cerebra, cerebella and hippocampi from young, 3 weeks and aged, 24 month old rats, respectively. Each group comprised 4–7 animals. Brain tissue extracts of each animal were prepared and protein as well as proteasome contents were measured. As shown in Table 1 the higher weight of the brain parts of aged as compared to young rats went along with an appropriate higher content in total protein. Interestingly, despite this age-dependent rise in protein content there was no parallel increase in the amount of proteasome. Thus, the percentage of proteasome in total brain protein was lower in the brain parts of aged as compared to young rats, namely −19% in cerebrum, −31% in cerebellum and −37% in hippocampus (Table 1).

**Table 1:** Tissue weight, total protein and proteasome content.

	tissue weight	total protein	proteasome	
	(g)	(mg)	(µg)	(% of total protein)
Cerebrum				
Young	1.07±0.08	19.6±1.3	393.0±18.5	2.00
Aged	1.50±0.05	26.2±0.9	402.8±24.8	1.63
p<	0.001*	0.009*	0.841	
Cerebellum				
Young	0.34±0.01	7.5±0.3	124.6±11.4	1.65
Aged	0.49±0.08	11.2±0.7	128.5±15.2	1.14
p<	0.007*	0.001*	0.843	
Hippocampus				
Young	0.068±0.003	1.8±0.1	32.4±4.0	1.77
Aged	0.110±0.027	2.6±0.3	29.3±6.2	1.12
p<	0.025*	0.051	0.688	

Means ± SEM and p values are given for comparison of young and aged rats. Number of animals per age group used were 5 for cerebrum, 7 for cerebellum, and 4 for hippocampus.

### Resolution of the proteasome spectrum in the brain areas of young and aged rats

**Figure 1:**
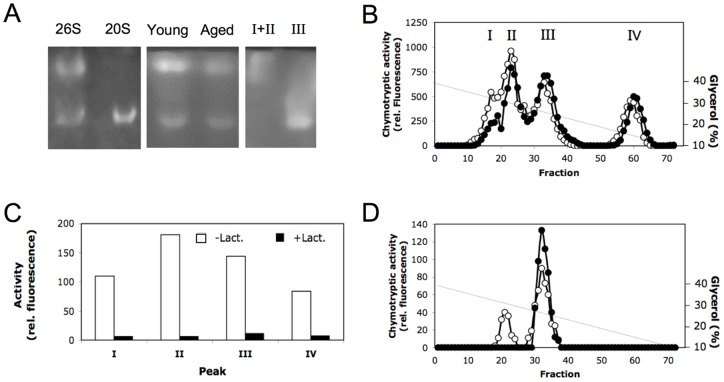
Detection and separation of proteasomes by non-denaturing polyacrylamide gel electrophoresis and glycerol gradient centrifugation. Panel A. Detection of chymotrypsin-like activity in polyacrylamide gels after non-denaturing electrophoresis by substrate overlay technique. Lanes 26S and 20S each contain 2 µg of purified erythrocyte 26S and 20S proteasome, respectively. Lanes Young and Aged contain 170 µg of tissue extracts each of cerebrum from young and aged rats; an aliquot of pooled fractions of peak Ι and ΙΙ as well as ΙΙΙ obtained after glycerol gradient centrifugation of rat cerebrum were run in lanes Ι+ΙΙ and ΙΙΙ, respectively. Panel B. Extracts of brain tissue from young (open circles) and aged (filled circles) rats were separated by centrifugation in a glycerol gradient (40–10%) and then fractionated. Each fraction was tested for its content of chymotrypsin-like activity. Panel C. Chymotrypsin-like activity in the four peaks (I, II, III, IV) obtained by glycerol gradient centrifugation was measured in the presence (black columns) and absence (white columns) of the 50 µM lactacystin. Panel D. 30 µg each of 20S proteasome (filled circles) and 26S proteasome (open circles) purified from human erythrocytes were subjected to glycerol gradient centrifugation under the same conditions as described in panel B and their chymotrypsin-like activity was measured.

When tissue extracts of cerebrum were subjected to non-denaturing PAGE and proteasome activity detected in-gel by substrate overlay, two different proteolytically active bands could be observed in brain extracts of young and aged rats and their fluorescence has been quantified (Fig. 1, lane Young and Aged, respectively). They corresponded to 20S proteasomes and 26S proteasomes as revealed by a parallel run of 20S and 26S proteasomes purified from human erythrocytes (Fig. 1A). For 26S proteasomes from young and aged rats (means ± SEM; n = 4) the relative fluorescence was 82.3±11.4 and 37.6±2.4, respectively. For 20S proteasomes (n = 3) the relative fluorescence were 27.3±3 and 17.7±2.9 for young and aged rats, respectively, indicating that in aged animals the 26S and 20S proteasome activities were 55% and 36% lower than in young animals. Since equal amounts of tissue extract protein from young and aged animals were subjected to electrophoresis, the lower proteasome activities in aged as compared to young rats could be due to the lower proteasome concentration or to lower specific activity of the proteasome complexes in aged rats. Therefore, to determine the specific proteasome activity in brain tissue of young and aged rats the enzymes have to be isolated and quantified before activity determination.

Therefore, we applied brain extracts to centrifugation on 10%–40% glycerol gradients. The total Suc-LLVY-MCA hydrolysing activity could be separated into four peaks sedimenting at about 32%, 30%, 25%, and 15% of glycerol, respectively (Fig. 1B). To determine which of the four activity peaks contained 20S and 26S proteasomes, Suc-LLVY-MCA hydrolysing activity was also determined in the presence of the specific proteasome inhibitor lactacystin. As shown in Fig. 1C the activity of all four peaks was inhibited by lactacystin to more than 90%.

To identify which of them actually contained proteasomes we centrifuged 20S and 26S proteasomes purified from human erythrocytes on glycerol gradients under the same conditions for comparison. As shown in Fig. 1D 26S proteasomes split up into two peaks appearing in fractions 20–25 and fractions 30–35. Analysis of the protein content of both peaks by non-denaturing PAGE revealed that the 26S proteasome preparation also contains 20S proteasome, that sedimented in fractions 30–35, while the 26S proteasome sedimented in fractions 20–25 of the gradient (Fig. 1A, lane 26S and 20S). Analysis of the activity peaks obtained by centrifugation of cerebrum extract by native PAGE showed that peak III contained 20S proteasome and peak I/II contained 26S proteasome, which is known to appear in a 26S form (20S associated with one 19S regulator, peak II) and in a 30S form (peak I) due to the association of two 19S regulators (Fig. 1A, lane I+II and III). Peak IV did not contain any proteasome complexes.

Although, the identity of the enzyme activity in peak IV remains to be worked out chromatographic analysis of the protein moiety on Superose 6 revealed the presence of a 105 kD protease (data not shown) that also shows hydrolysis of Suc-LLVY-MCA and is inhibited by lactacystin (Fig. 1B/C). An enzyme with properties like this has already been described to exist in human brain [16]. This result clearly shows that, in order to obtain reliable data on proteasome activity in brain extracts by measurement of the hydrolysis of Suc-LLVY-MCA, the prior separation of this 105 kD protease is absolutely essential.

### Amount and activity of 26S and 20S proteasomes in cerebrum, cerebellum and hippocampus of young and aged rats

To analyse the different proteasome populations of the three parts of the brain of young and aged rats with respect to their quantity and proteolytic activity, the brain extracts of each animal were applied to glycerol gradient centrifugation. Since a complete separation of 30S and 26S proteasomes was not possible by glycerol gradient centrifugation under the conditions used, we pooled all fractions comprising peak I and II for characterization of 26S/30S proteasomes and designated this subpopulation ‘26S proteasome’ (see Fig. 1). Fractions comprising peak III in the glycerol gradient were pooled for quantitative analysis of 20S proteasomes.

After separation by glycerol gradient centrifugation the amounts of 20S and 26S proteasomes were added up (µg proteasome/tissue; see Table 2). A comparison with the amounts measured in total tissue extracts (Table 1) revealed a recovery between 90% and 98%, except for aged cerebellum where it was only 78%. In all three parts of the brain the 26S proteasome comprises about 60–75% of the total amount of proteasomes. The proportion of both proteasome forms –20S as well as 26S – was not significantly different between young and aged animals (Table 2).

**Table 2:** Content of 20S and 26S proteasome in cerebrum, cerebellum, and hippocampus of young and aged rats.

	Proteasome content (µg/tissue)					
	Cerebrum		Cerebellum		Hippocampus	
	20S	26S	20S	26S	20S	26S
Young	102±24	285±40	39±4	81±10	6±1	23±2
Aged	103±35	282±26	31±4	69 ±11	8±2	20±3
p<	0.9820	0.960	0.187	0.469	0.548	0.530

Means ± SEM and p values are given for comparison of young and aged rats. Number of animals per age group used were 5 for cerebrum, 7 for cerebellum, and 4 for hippocampus.

**Figure 2:**
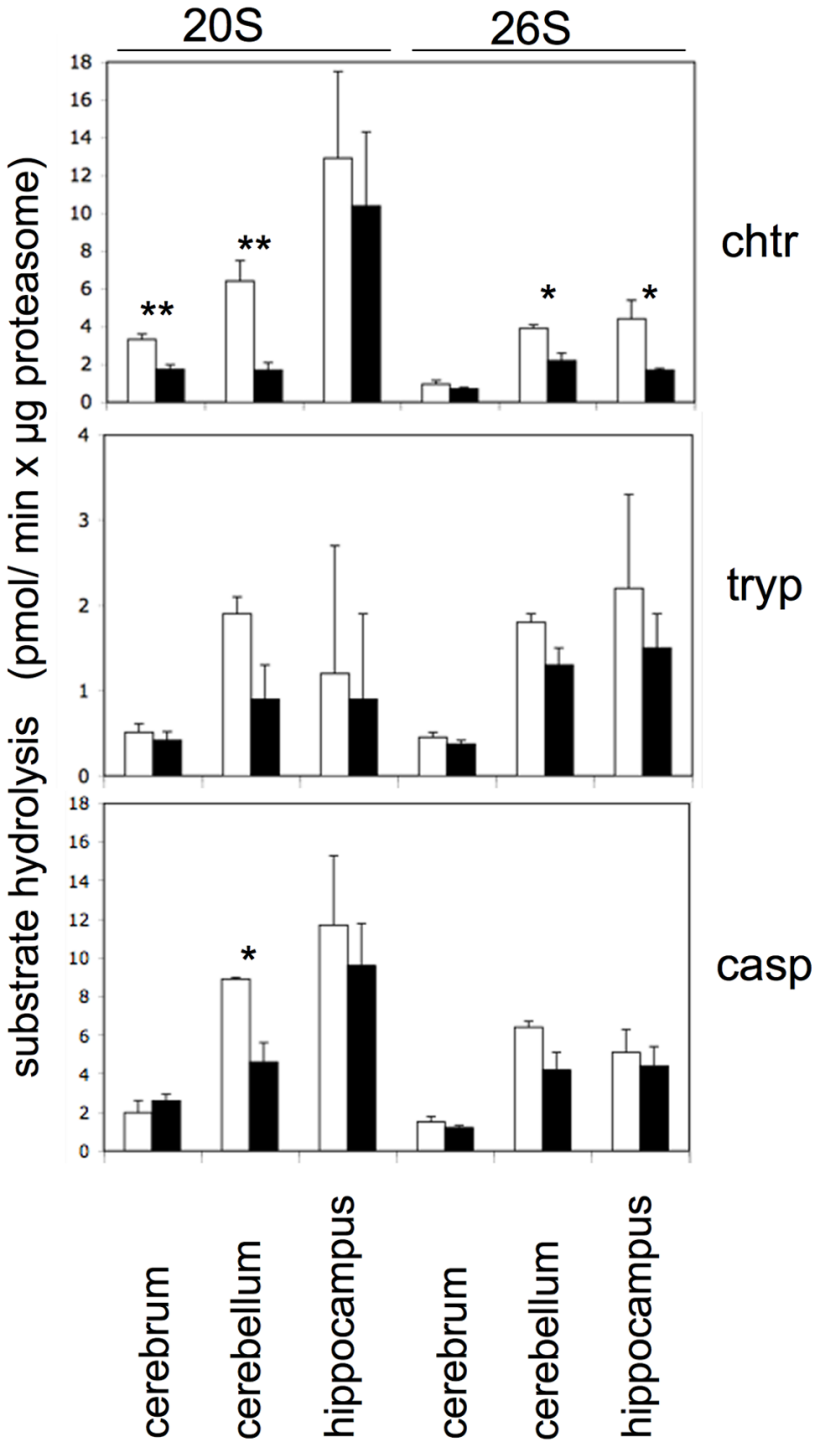
Specific hydrolytic activities towards fluorogenic peptide substrates of 20S and 26S proteasomes purified from different brain parts of young and aged rats. From cerebrum, cerebellum, and hippocampus of young (white columns) and aged (black columns) rats 20S and 26S proteasomes were separated by glycerol gradient centrifugation. The proteasome containing fractions were pooled and their proteasome content determined by immunoelectrophoresis. Chymotrypsin- (chtr), trypsin- (tryp) and caspase-like (casp) proteasome activities were measured and shown as pmol substrate hydrolysis/min x µg proteasome. Values are given as means ± SEM (n = 5) and data obtained for young and aged rats were compared by Students t-test. p-values indicating statistically significant differences are indicated (*, p<0.05; **, p<0.01).

Since glycerol gradient centrifugation led to the separation of the 105 kD protease and to a resolution of 20S and 26S proteasomes, their specific activities, i.e. activity per µg of 20S and 26S proteasomes, could now be determined using fluorogenic tripeptide substrates and were generally found to be lower in the brains of aged as compared to young rats (Fig. 2). However, despite this tendency only the decrease of the chymotrypsin-like activity of 20S proteasomes in cerebrum and cerebellum and of 26S proteasomes in cerebellum and hippocampus reached statistical significance. The trypsin-like and caspase-like activities of 20S and 26S proteasomes were also diminished in the brain components of aged rats but due to high individual variations did not reach statistical significance with the exception of the 20S proteasome caspase-like activity in cerebellum (Fig. 2).

Specific activities of the proteasomes in the different brain areas of young and aged rats differed not only in absolute terms but also with regard to the ratio of chymotrypsin-like to caspase-like activity. Independent of age and except of cerebal 20S proteasome of young animals the specific activities of 20S and 26S proteasomes from the cerebrum and cerebellum were generally the highest towards the caspase-specific substrate and the lowest towards the trypsin-specific substrate. This enzyme property was less pronounced or even absent in proteasomes from hippocampus (Fig. S1). In summary, these data show that proteasomes from the different parts of brain are functionally not identical and that during the aging process molecular alterations affecting proteasome activities proceed differently.

### Presence of immunosubunits augments in aging rat brain

**Figure 3:**
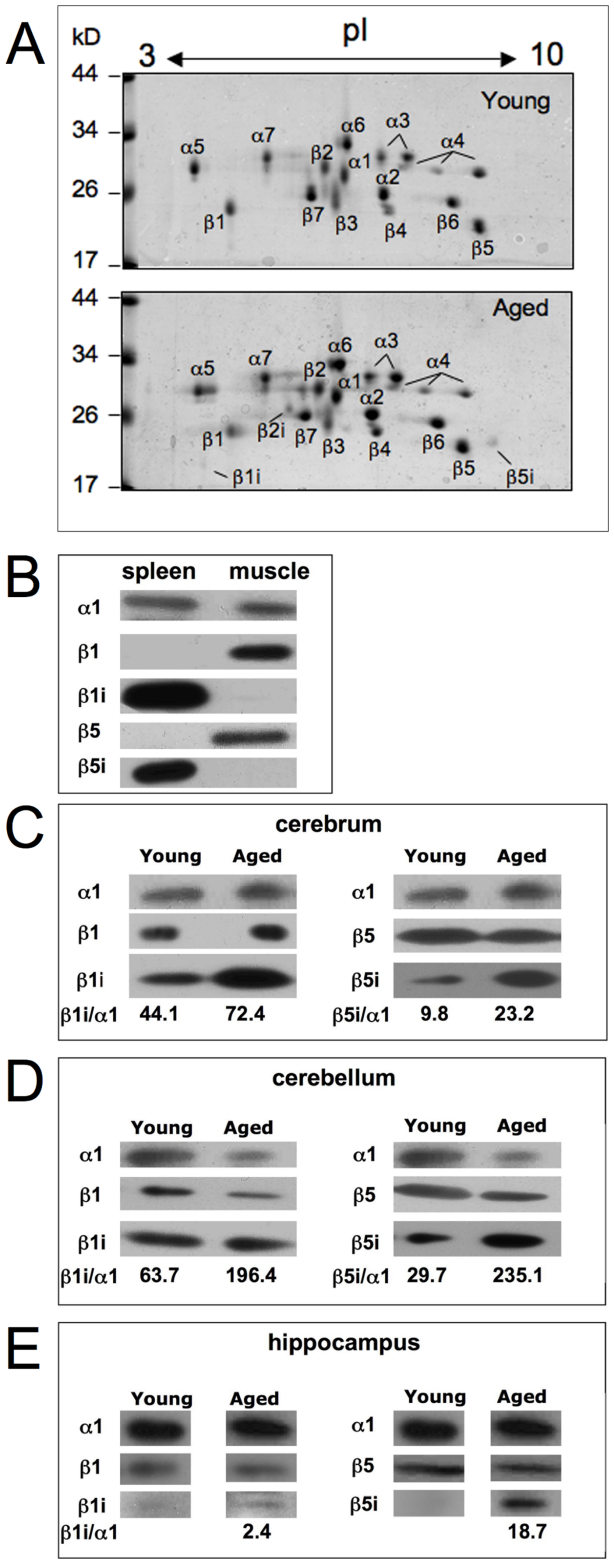
2D-PAGE electrophoresis of 20S proteasome purified from cerebellum of young and aged rats. Panel A. 30 µg of purified proteasome was applied to each gel, which were stained with Coomassie. Proteasome subunits were assigned according to our earlier investigations with proteasomes from rat liver [31]. The location of subunits β1i, β2i and β5i are indicated by a bar. Panel B–E. Standard- (β1 and β5) and immuno-subunits (β1i and β5i) in 26S proteasomes isolated from cerebrum (panel C), cerebellum (panel D), and hippocampus (panel E) from young and aged rats were detected by immunoblot analysis after SDS-PAGE. About 2–5 µg of 26S proteasome was subjected to the electrophoresis gels. The specificity of the antibodies was tested with 0.5 µg 20S proteasomes purified from rat spleen and muscle (panel B). As we are not aware of an antibody specific for rat proteasome β2 and β2i subunits, these proteins were not analysed here. As a loading control subunit α1 was identified in panel B–E and in panel C–E; the ratio of the signals (pixel intensity) of the immunosubunits β1i and β5i were calculated against α1 after their densitometric quantification by use of the ImageJ software.

To explore whether these changes of activity went along with alterations in the presence of standard- and immuno-proteasomes, we subjected material from the 20S and 26S proteasome pools to further purification by means of anion exchange chromatographies and gel filtration. Since proteasomes from cerebellum had shown the clearest changes in activity, they were applied to analysis by 2D-PAGE analysis. Except of one investigation [17] proteasomes purified from brain tissue of different mammals, like rat, cow and humans [18]–[24] were always reported to contain standard proteasomes, only. The results of our investigation confirmed these published data for the brains of young rats. However, careful inspection of the subunit pattern obtained with material from aged rats revealed also faint spots of the immunosubunits β1i, β2i and β5i (Fig. 3A).

This result prompted us to inspect proteasomes purified to apparent homogeneity from all three parts of brain by means of SDS-PAGE and immunoblotting with antibodies to subunit β1, β1i, β5, and β5i. To ascertain the specificity of the antibodies used in this investigation they were tested against 20S proteasomes purified from rat skeletal muscle (subtype I–III) and spleen, which contain standard- and immuno-proteasomes, respectively [9] (Fig. 3B). By probing high concentrations (3–5 µg) of the brain proteasomes we detected β1i and β5i subunits in cerebrum and cerebellum of young and aged rats (Fig. 3C, D), whereas only minor amounts of β1i and β5i could be detected in young hippocampus (Fig. 3E). We have densitometrically quantified the blot signals by means of the ImageJ software and calculated the ratios of immunosubunits against subunit α1 monitored by immunoblotting with the α1-specific antibody IB5. Since in hippocampus of young rats immunosubunits were almost invisible, the ratio against α1 was not calculated in this case. In all brain areas we observed an increase in the amount of immunosubunits in aged rats (Fig. 3C, D, E), thereby suggesting an increase in the amount of immuno- or intermediate-type proteasomes during the aging process.

### Poly-Ub-protein degradation capacity by 26S proteasomes is not impaired during aging

Degradation of poly-ubiquitinated proteins by 26S proteasomes requires their binding, deubiquitination and unfolding by the 19S regulator before the substrate protein can reach the central proteolytic cavity of the 20S core particle. Because the last two steps are not required for degradation of fluorogenic peptide substrates, peptide-hydrolysing activities determined with these substrates may not reflect the actual, physiologically more relevant activity of 26S proteasomes.

**Figure 4:**
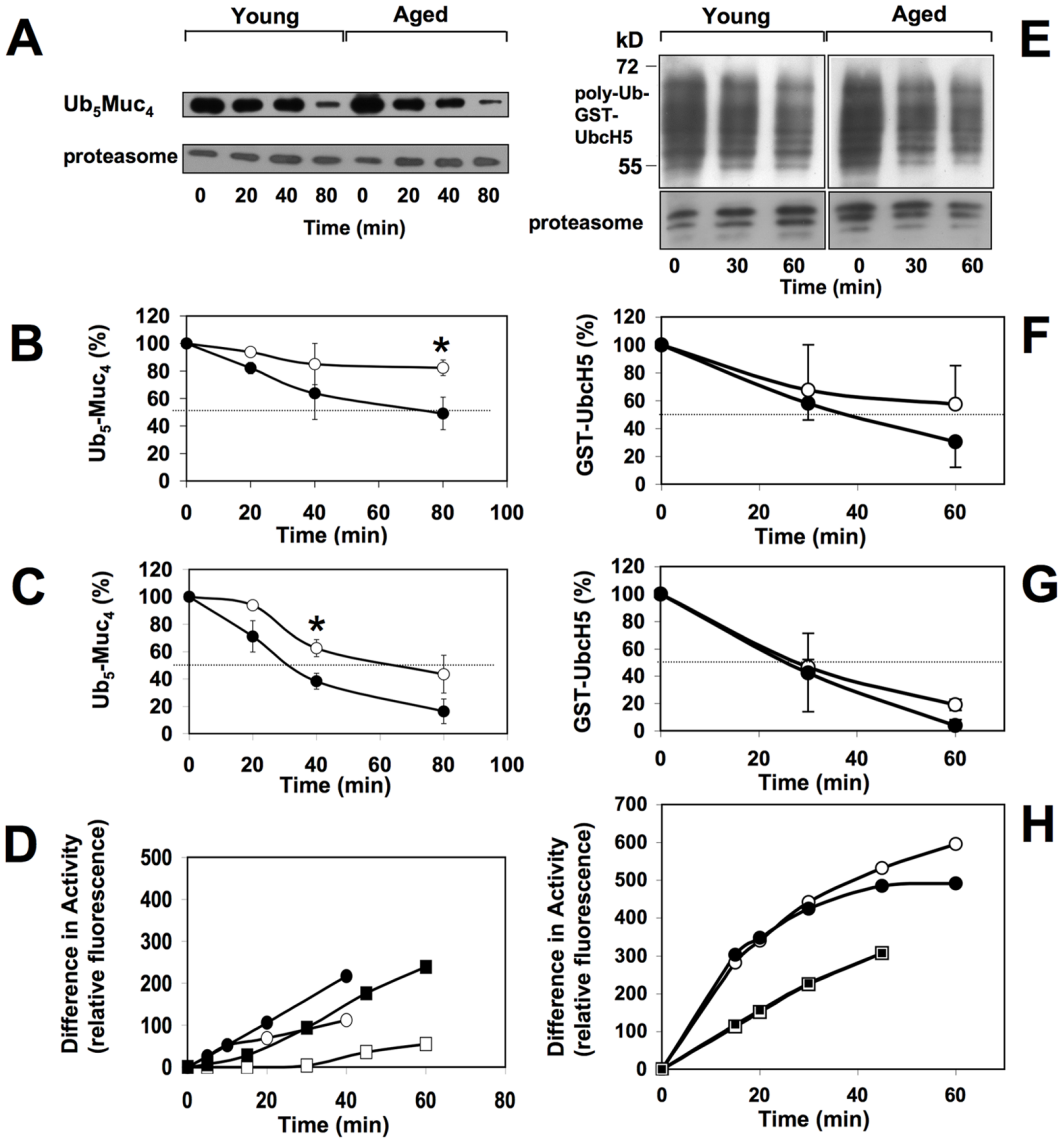
Degradation of Ub_5_Muc_4_ and poly-Ub-GST-UbcH5 by 26S proteasomes purified from cerebrum and cerebellum. Panel A–D. 70 nM of purified 26S proteasomes from young and aged animals were incubated with 600 nM Ub_5_Muc_4_ and incubated at 37°C. At the times indicated aliquots of the reaction mixture were removed and subjected to SDS-PAGE. Afterwards Ub_5_Muc_4_ was detected on immunoblots with an antibody raised against Muc_950–958_ peptide. Panel A shows a representative result of the experiments performed. Proteasome subunit α1 was monitored as a loading control with antibody IB5. For quantitative determination of the degradation process, the amount of Ub_5_Muc_4_ on the blots was measured by densitometry. Results obtained are indicated for the digestion processes with 26S proteasomes from cerebrum (panel B) and cerebellum (panel C) of young (open circles) and aged (filled circles) rats. Data shown are means ± SEM of 3–5 animals. Values of the degradation rates were compared between young and aged animals and statistically different values (*, p<0.05) are indicated. Panel D. 26S proteasome (30 nM) purified from cerebellum (circles) and cerebrum (squares) of young (white symbols) and aged (black symbols) rats were preincubated with or without 300 nM Ub_5_Muc_4_ for 15 min at 37°C before 100 µM Suc-LLVY-MCA was added and fluorogenic peptide hydrolysis was measured at the indicated time points. The data (means of two animals) shown are the difference of hydrolytic activity measured after preincubation with and without Ub_5_Muc_4._ Panel E–H. Purified 26S proteasome (30 nM) from young and aged animals was incubated with 800 nM poly-Ub-GST-UbcH5 at 37°C. After 30 and 60 minutes aliquots of the reaction mixtures were removed and subjected to SDS-PAGE, blotted and UbcH5 was detected on immunoblots using the Ubch5 antibody. Panel E shows a representative result of the experiments performed. Proteasome was monitored as a loading control by using the 20S proteasome antiserum 37. For quantitative determination of the degradation process, the amount of poly-Ub-GST-UbcH5 on the blots was measured by densitometry. Results obtained are indicated for the digestion processes with 26S proteasomes from cerebrum (panel F) and cerebellum (panel G) of young (open circles) and aged (filled circles) rats. Data shown are mean values ± S.D. of 2 animals. Panel H. 26S proteasome purified from cerebellum (circles) and cerebrum (squares) of young (white symbol) and aged (black symbol) rats were preincubated with or without poly-Ub-GST-UbcH5 (same ratio as detailed for panel E–G) for 15 min at 37°C before 100 µM Suc-LLVY-MCA was added and fluorogenic peptide hydrolysis was measured at the indicated time points. Data shown are representative of two independent experiments.

To measure the 26S proteasome-catalysed degradation of an ubiquitinated substrate we used a linear tetramer of a nonapeptide from the sequence of the mucin glycoprotein MUC1 that was N-terminally fused to ubiquitin, which again was conjugated to tetra-ubiquitin at lysine-48, abbreviated Ub_5_Muc_4_. In a previous study we had shown that this substrate is degraded by purified 26S proteasomes in an ATP-dependent manner [12]. Here we incubated Ub_5_Muc_4_ with equal amounts of 26S proteasomes (monitored by immunoblotting with antibody IB5 to proteasome subunit α1) chromatographically purified from cerebrum and cerebellum of young and aged rats (Fig. S3) and measured the substrate degradation by means of immunoblotting as described previously [12] (Fig. 4A). Cerebral 26S proteasomes of aged rats exhibited an approximately 2.7-fold increased poly-Ub-substrate degradation activity when compared with the corresponding 26S proteasomes of young rats. Whereas the 26S proteasomes from aged rats had degraded 50% of the substrate after 80 min, the enzyme of young animals degraded only 18% within the same period of time (Fig. 4B). 26S proteasomes from cerebellum exhibited, independent of age, a considerably higher poly-Ub-substrate degradation activity than cerebral 26S proteasomes. Nevertheless, 26S proteasomes from cerebellum of aged rats also possessed an approximately 2-fold increased poly-Ub-substrate degradation activity compared with 26S proteasomes from young rats (Fig. 4C).

To substantiate this finding we additionally used poly-ubiquitinated GST-tagged UbcH5 as a substrate. This GST-tagged ubiquitin-conjugating (E2) enzyme can be isolated as an auto-poly-ubiquitinated protein when preincubated with purified E1 and ubiquitin [13]. As shown in Fig S4 this protein exists in various poly-ubiquitinated forms. There was no age-dependent difference in the rate of degradation of this substrate by 26S proteasomes from cerebrum and cerebellum (Fig. 4 E–G).

These data show that 26S proteasomes from aged rats despite their decreased activity towards short fluorogenic peptide substrates exhibited the same or even enhanced poly-Ub-substrate degradation activity when compared with 26S proteasomes of young rats.

Previously it was shown that binding of a poly-ubiquitinated substrate to the 26S proteasomes results in the activation of the proteasomal peptide hydrolysing activity [12], [25]. When we tested the effect of Ub_5_Muc_4_ substrate binding to 26S proteasomes isolated from cerebrum of young and aged animals a similar result was obtained. Strikingly, the peptide hydrolysing activity of 26S proteasomes of aged animals was stronger activated by binding of Ub_5_Muc_4_ substrate than 26S proteasomes of young animals (Fig. 4D). This activating effect was even higher when poly-Ub-GST-UbcH5 was used as a substrate, however with this substrate no difference was observed between 26S proteasomes from young and aged rats (Fig. 4H).

## Discussion

The process of aging most likely combines an intrinsic cellular senescence program with environmental effects potentially imposing harmful attacks on the organism. Aging also includes changes of the finely tuned cellular proteostasis that is based on the balance between protein biosynthesis and degradation. Any alteration of this balance may also affect the cellular protein degradation machinery. Thus, impairment of the protein degradation rate can result in an accumulation of proteins that may be non-functional any more or mis-folded. If not eliminated such defective proteins are prone to forming aggregates, which eventually will induce cell death, a mechanism proposed to be responsible for the development of neurodegenerative diseases.

In the present study we have analysed and compared the molecular composition and protein degradation activity of 20S/26S proteasomes in three different parts of the brain from young and aged rats. Our analyses show that proteasomes in young and aged animals are predominantly standard proteasomes but aging leads to an increased amount of immuno-subunits in a small fraction of proteasomes in the brain. Despite the fact that this is paralleled by a significant decrease in the specific activity towards short fluorogenic peptides, the capacity to degrade poly-ubiquitinated proteins reflecting more likely physiological substrates is conserved in the aged brain.

It has repeatedly been described that the proteasomal activity in brain declines with increasing age, albeit to different extents depending on which proteasome activity was determined and which brain regions were analysed [3], [6], [26]. In all of these studies proteasome peptidolytic activity was referred to tissue total protein quantity. However, estimation of proteasome activity in tissue extracts may be critical with regard to the reference value used and with respect to the fact that the substrate used may not only be degraded by the proteasome. This is in particular true for fluorogenic peptide substrates like Suc-LLVY-MCA and Bz-VGR-MCA, which are hydrolysed efficiently also by other intracellular proteases, like calpains and TPPII. In addition, in brain tissue a 105 kD protease has been detected that hydrolyses both fluorogenic peptide substrates and that is also inhibited by the specific proteasome inhibitor lactacystin [16]. To avoid the interference of this and possibly other proteinases that may obscure the measurement of proteasomal proteolytic activities, the brain tissue extracts were fractionated by glycerol-gradient centrifugation and applied to quantitative rocket immunoelectrophoresis, a procedure allowing to determine absolute proteasome amounts in complex protein mixtures and thus to calculate specific proteasome activities. This turned out to be of utmost importance because our investigation shows that the total content of proteasome per brain area was not different between young and aged rats. The proteasome amounts measured before and after separation of tissue extract by glycerol gradient centrifugation differed only by about 10% and was about 1.5% of total soluble protein, which is in agreement with earlier data published [27], [28]. In contrast, we found an age-dependent decrease in proteasome concentration of approximately 25%–38% due to the increase in total weight and total protein content when the three parts of brain of young and aged rats were compared. Since the number of glial cells is also increased in aging brain [29], a similar decrease of proteasome concentration would also be observed when referred to total DNA content. A decrease in the concentration of certain proteasome subunits in brain and spinal cord extracts of mice and rat of different ages was found also by other investigators [4], [26], [30].

Not only the separation of non-proteasomal proteolytic enzymes, e.g. the 105 kD protease, but also the resolution of different proteasome forms is a prerequisite for accurate measurement of specific proteasome activity in brain tissue. This was obtained by the glycerol gradient centrifugation step allowing to observe a drop from young to aged animals of especially the specific (referred to absolute proteasome amount) chymotrypsin-like activity of 20S and 26S proteasomes. Overall, all three proteasomal activities measured in the three brain parts of aged animals were lower as compared to young rats, but especially the chymotrypsin-like activity reached statistical significance.

Since we can exclude that this loss of activity is due to a decrease in the absolute amount of 20S and 26S proteasomes in the brain of aged animals, the observed alterations must originate from intrinsic changes of the enzymes. Our investigations show that the pattern of 20S proteasome subtypes changes from young to aged rats in cerebrum and in cerebellum (see Fig. S2). As we have shown before that 20S proteasome subtypes differ in their subunit composition and their proteolytic activity [9], [31], dynamic age-dependent alterations of proteasomes take place in the brain. These may also go along with de novo proteasome biogenesis, since Dasuri and colleagues found an increased signal for the proteasome assembly chaperone in aged brain and concluded that there is an increased proteasome biogenesis during aging [30].

Proteasomes from brain tissue were described to be almost exclusively of standard subunit composition. However, under certain conditions, like Huntington disease, Alzheimer disease, multiple sclerosis and temporal lobe epilepsy as well as in animal models for amyotrophic lateral sclerosis immunosubunits were detected in cerebral and spinal cord tissue, respectively [32]–[34]. Similarly, subunit β1i was detected in human hippocampus in non-demented elderly, but not in young controls [8]. An age-related increase in immunoproteasome content was also described for rat hippocampus [35] and muscle [7]. Our Coomassie-stained 2D-gels revealed that standard β-subunits were still predominant, however, small amounts of the immuno-subunits β1i, β2i and β5i appeared in 20S proteasomes from aged cerebellum. In fact, our immunoblot analyses revealed that albeit to a very low concentration immunosubunits are already present in brain proteasomes from young rats and their concentrations seem to increase in proteasomes from aged rats. Similar to brain tissue the majority of proteasomes in skeletal muscle contain standard subunits and only a small amount are proteasomes of immuno- and intermediate-type. As the latter two groups were found to have lower specific activities towards fluorogenic peptides substrates than standard-proteasomes [7], [9], the increase in brain of proteasomes containing immuno-subunits at least partly explains the age-dependent drop of the proteasomal chymotrypsin- and caspase-like activity. Although small differences within the substrate binding site (S1 pocket) within subunits β5 and β5i were observed in mice proteasomes [36], the molecular reason underlying the lower specific activity of proteasomes from aged rats is not known so far. As observed on the 2D-gels (Fig. 3A) there are also small differences in the pattern of subunits not directly involved in active site formation. The most prominent is the double spot of subunit α5 in aged rats present in young rats just as a single spot. In yeast proteasomes this subunit was found to be partially N-acetylated, a modification that could explain the existence of a double spot. However, there is no evidence that this posttranslational modification affects the proteasomal activity [37].

We have not analysed whether the small amount of immunoproteasomes are located in neurons or in microglia, astrocytes and endothelial cells that are known to express immunosubunits under certain conditions [8], [38], [39]. An age-dependent increase in immunoproteasomes could result from a gain in the number of microglia and astrocytes compared to neurons as has been found in aging mice [29]. This phenomenon may be part of the process called inflamm-aging, a process of continuous but restricted inflammation leading to tissue damage and eventually to age-related diseases like neurodegeneration [40]).

Oxidative damage of cellular compounds including the proteasomal system has often been related with the aging process [41]. Especially the 26S proteasome has been described as vulnerable by oxygen radicals while the 20S proteasome has been found to be relatively stable. This could be of importance as it was postulated that the removal of oxidatively damaged proteins is predominantly performed by the 20S proteasome [42]. We obtained no evidence for a selective loss of the activity of 26S proteasomes towards fluorogenic peptide substrates. Quite in contrast, testing the enzyme activity with the previously described poly-Ub-substrate Ub_5_-(MUC)_4_ 26S proteasomes from cerebrum and cerebellum from aged rats exhibited an about two-fold increased substrate degradation rate as compared to 26S proteasomes from brains of young animals. Since another substrate, poly-Ub-GST-UbcH5, was degraded at about the same rate by the enzymes from aged as compared to young rats, we conclude that the capacity to degrade poly-Ub-substrates does not decline with age and determination of activities with fluorogenic peptide substrates might not reflect the actual activity towards poly-Ub-protein substrates.

Hydrolysis of peptide bonds occurs within the central proteolytic chamber of the 20S proteasomes. Access to this chamber occurs through the substrate entrance pore in the outer α-rings of the 20S proteasome. Fluorogenic tripeptide substrates can easily enter the central chamber as long as the pore gate is open, which is routed by binding of regulators like PA28 and 19S regulator. On the other hand, the processing by 26S proteasomes of poly-Ub-substrates is much more complex, since these substrates have to be recognized, bound and deubiquitinated by 19S regulator subunits before being transferred into the central proteolytic chamber [43], [44]. Therefore, it may not be unlikely that the degradation rates of small fluorogenic tripeptide substrates are different from those for poly-Ub-protein substrates. We have found no difference in the ratio of 19S regulator lid (Rpt 4 and Rpt6) and base (Rpn 1, Rpn 2, Rpn 11) subunits to 20S proteasome subunits in 26S proteasomes from aged as compared to young rats (Fig. S3 B and C), indicating that the ratio of 19S regulator to 20S proteasome is not different between young and aged rats. We have also found no age-related difference in the 26S proteasome associated deubiquitinating enzyme USP14 (data not shown) as was recently described for T cell proteasomes obtained from elderly donors [45]. In accordance with our results Cook and coworkers [46] also detected no impairment of 26S proteasome activity in brain with increasing age. In a previous study it was shown that the incorporation of immunosubunits resulted in a higher degradation activity versus poly-ubiquinated substrates by 26S proteasomes being essential for the elimination of oxidized poly-ubiquitinated proteins and the prevention of protein aggregate formation in the brain under inflammatory conditions [47]. Similarly, in cardiomyocytes lacking the proteasomal immunosubunits accumulation of poly-Ub-substrates and decreased activation of NFκB was found when the cells were treated with IFNγ [48]. Thus the higher content of immunosubunits in 26S proteasomes from brain of aged rats (Fig. 3 C–E) may contribute to their (enhanced) degradative capacity of poly-Ub substrates.

Since oxidatively modified proteins can be substrates for the ubiquitination machinery [47], [49], [50], a conserved or even accelerated activity of 26S proteasomes towards proteins might well be supportive in coping with oxidant damaged proteins that were found to increase in rat brain under normal aging conditions [5] and therefore may be a prerequisite for an uncomplicated aging process [51]–[53]. One might speculate that the alteration of different metabolic pathways and their products (*e.g*. oxidized proteins) during brain aging triggers the increase of 26S immunoproteasome content and 26S proteasome activities thereby facilitating the clearance of poly-Ub-conjugates. When this essential function of UPS is, for unknown reasons, affected in aged brain protein aggregates might form leading to the appearance of neurodegenerative diseases as observed for instance in hippocampus of rats after induction of neuroinflammation and proteasome inhibition [54].

In conclusion, our data reveal that in a dynamic process proteasome composition and activity changes upon aging. We show for the first time that the degradative capacity for ubiquitin-conjugated substrates is not impaired during aging. This phenomenon may reflect a basic requirement for an undisturbed aging process under non-pathological conditions.

## Supporting Information

Figure S1 **Specific peptide hydrolysing activities of 20S and 26S proteasomes from cerebrum, cerebellum and hippocampus.** 20S and 26S proteasomes were isolated by glycerol gradient centrifugation from cerebrum, cerebellum and hippocampus of young and aged rats. After the proteasome content was determined by means of immunoelectrophoresis their hydrolytic activity was measured towards fluorogenic peptide substrates to determine their specific chymotrypsin-like (grey bars), trypsin-like (black bars), and caspase-like (white bars) activity. Data are means ± SEM (n = 5) and from the same experiments as shown in Fig. 2. Values of chymotrypsin-like vs. trypsin-like, trypsin-like vs. caspase-like and chymotrypsin-like vs caspase-like activities were compared by Students t-test. P-values indicating statistically significant differences are indicated (*, p<0.05; **, p<0.01; ***, p<0.001). Comparisons with p>0.05 are indicated without star.
(TIF)

Figure S2 **Subtype patterns of 20S proteasomes purified from cerebrum (A) and cerebellum (C) of young (dashed line) and aged (solid line) rats.** Separation of the different subtypes (I, II, III, IV, V) was performed by high-resolution anion exchange chromatography on a Mini Q column by a linear increasing concentration of NaCl [9]. Only the sections between 295–340 mM NaCl of the chromatograms are shown. The chymotrypsin-like activity was measured in fractions of panel A and C and pictured in panel B (cerebrum) and D (cerebellum). White circles represent the activity of proteasomes from of young, black circles the activity of aged rats.
(TIF)

Figure S3 **Non-denaturing and SDS-PAGE of 26S proteasomes purified from brain tissues.** Panel A. From young and aged rats 4 µg each of 26S proteasome purified from cerebrum and 4 µg each of 26S as well as 20S proteasomes purified from cerebellum were subjected to non-denaturing polyacrylamide gel electrophoresis and the gels stained with Coomassie blue. As references 26S proteasome purified from rat liver (4 µg) and 20S proteasome purified from human erythrocytes (3 µg) were used. Panel B. 2 µg each of 26S proteasome purified from cerebrum and cerebellum of young and aged rats was subjected to SDS polyacrylamide gel electrophoresis and the gels stained with Coomassie blue. Molecular mass standards of 17–170 kD were run in parallel. Panel C. The protein amounts of 19S regulator subunits (indicated according to Shibatani et al 2006) and a 20S proteasome subunit (27 kD) in panel C were densitometrically measured in cerebral 26S proteasomes by the ImageJ software and the ratio of 19S Reg subunits to the 27 kD was calculated. The data show similar ratios for 19S Reg/20S proteasome in 26S proteasomes from young and aged rats. Similar data were obtained for cerebellar 26S proteasomes (data not shown).
(TIF)

Figure S4 **Stability of Ub_5_Muc_4_ and polyUb-GST-UbcH5 in the presence and absence of 26S proteasome.** Panel A. 600 nM Ub_5_Muc_4_ was incubated (at 37°C) with 60 nM 26S proteasome in the presence of 100 µM proteasome inhibitor MG132 (+26S/MG132), without 26S proteasome (−26S) and with 60 nM 26S proteasome (+26S) purified from human erythrocytes. At the times indicated aliquots of the reaction mixture were removed and subjected to SDS-PAGE. Afterwards Ub_5_Muc_4_ was detected on immunoblots with an antibody raised against Muc_950–958_ peptide. Panel B. Poly-Ub-GST-UbcH5 (0.5 µg/lane) was subjected to SDS-PAGE, blotted and then detected by use of antibodies to UbcH5 (lane 1), poly-Ub (lane 2), and GST (lane 3), respectively. Panel C. 800 nM polyUb-GST-UbcH5 was incubated with 60 nM 26S proteasome in the presence of 100 µM proteasome inhibitor MG132 (+26S/MG132), without 26S proteasome (−26S) and with 60 nM mM 26S proteasome (+26S) from human erythrocytes at 37°C. At the times indicated aliquots of the reaction mixture were removed and subjected to SDS-PAGE and remaining poly-Ub-GST-UbcH5 was detected on immunoblots with an antibody raised against UbcH5.
(TIF)
