# Supplementary figures and images for: Poly-Ub-Substrate-Degradative Activity of 26S Proteasome Is Not Impaired in the Aging Rat Brain
Source: PLoS One. 2013 May 7;8(5):e64042. doi: 10.1371/journal.pone.0064042 (PMC3646778; doi:10.1371/journal.pone.0064042)

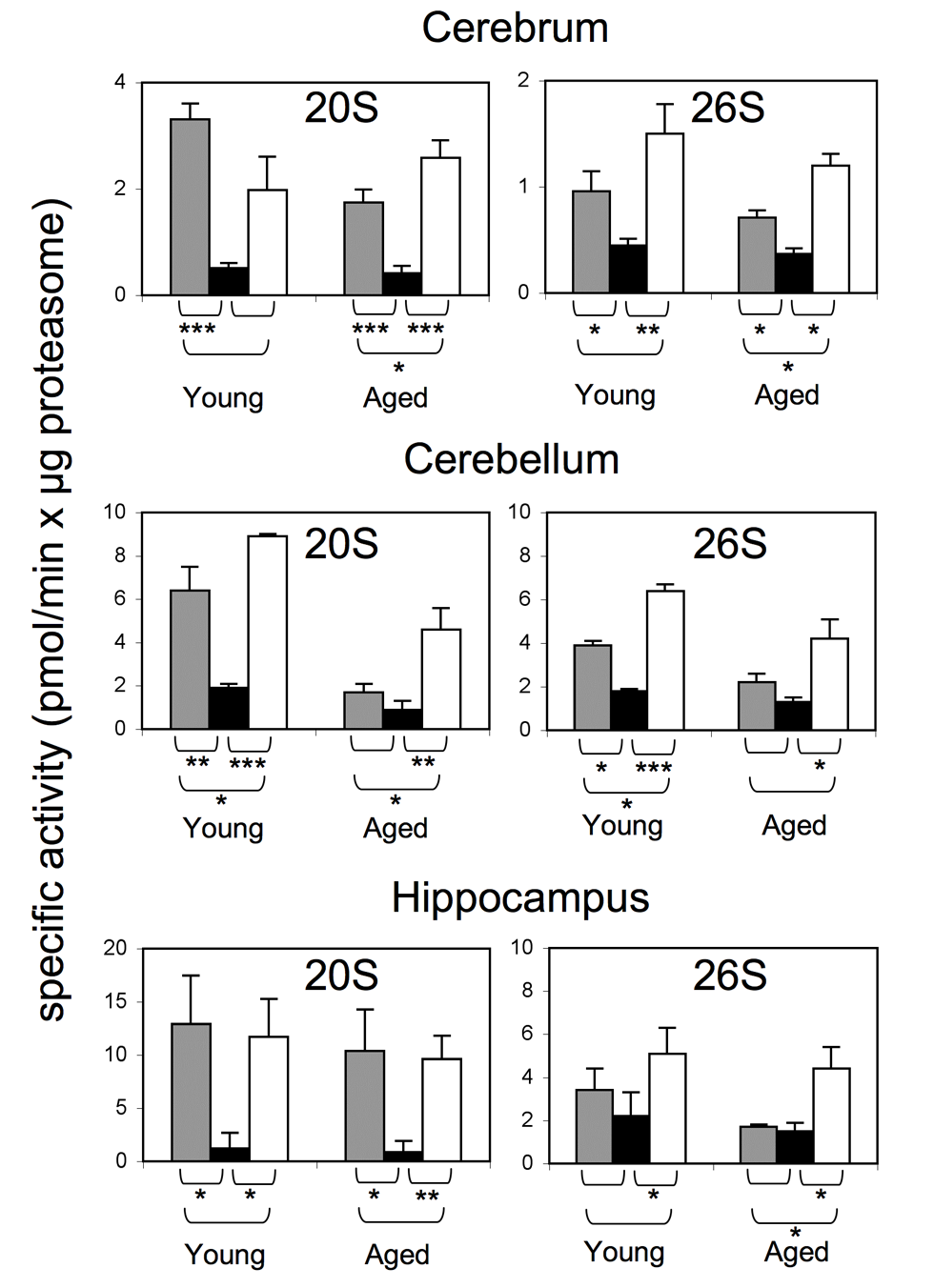

Supplement: Figure S1 — Specific peptide hydrolysing activities of 20S and 26S proteasomes from cerebrum, cerebellum and hippocampus. 20S and 26S proteasomes were isolated by glycerol gradient centrifugation from cerebrum, cerebellum and hippocampus of young and aged rats. After the proteasome content was determined by means of immunoelectrophoresis their hydrolytic activity was measured towards fluorogenic peptide substrates to determine their specific chymotrypsin-like (grey bars), trypsin-like (black bars), and caspase-like (white bars) activity. Data are means ± SEM (n = 5) and from the same experiments as shown in Fig. 2. Values of chymotrypsin-like vs. trypsin-like, trypsin-like vs. caspase-like and chymotrypsin-like vs caspase-like activities were compared by Students t-test. P-values indicating statistically significant differences are indicated (*, p<0.05; **, p<0.01; ***, p<0.001). Comparisons with p>0.05 are indicated without star. (TIF) [file pone.0064042.s001.tif]

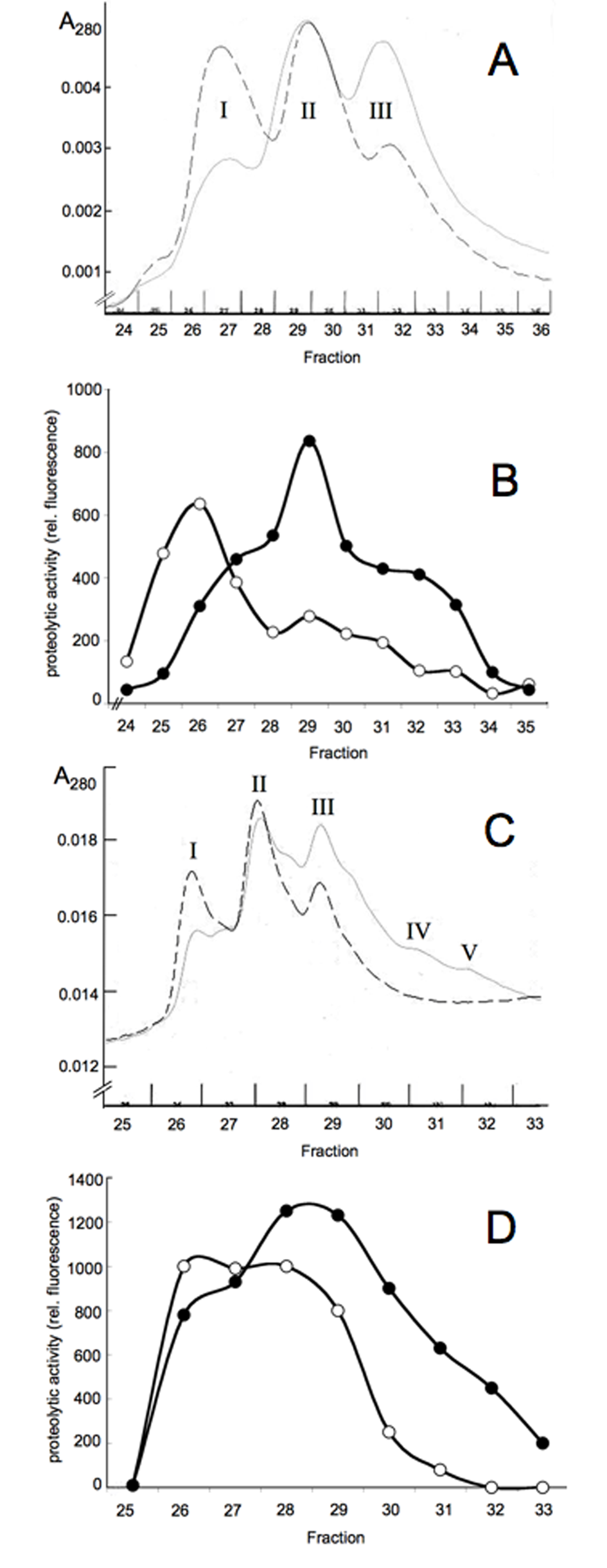

Supplement: Figure S2 — Subtype patterns of 20S proteasomes purified from cerebrum (A) and cerebellum (C) of young (dashed line) and aged (solid line) rats. Separation of the different subtypes (I, II, III, IV, V) was performed by high-resolution anion exchange chromatography on a Mini Q column by a linear increasing concentration of NaCl [9]. Only the sections between 295–340 mM NaCl of the chromatograms are shown. The chymotrypsin-like activity was measured in fractions of panel A and C and pictured in panel B (cerebrum) and D (cerebellum). White circles represent the activity of proteasomes from of young, black circles the activity of aged rats. (TIF) [file pone.0064042.s002.tif]

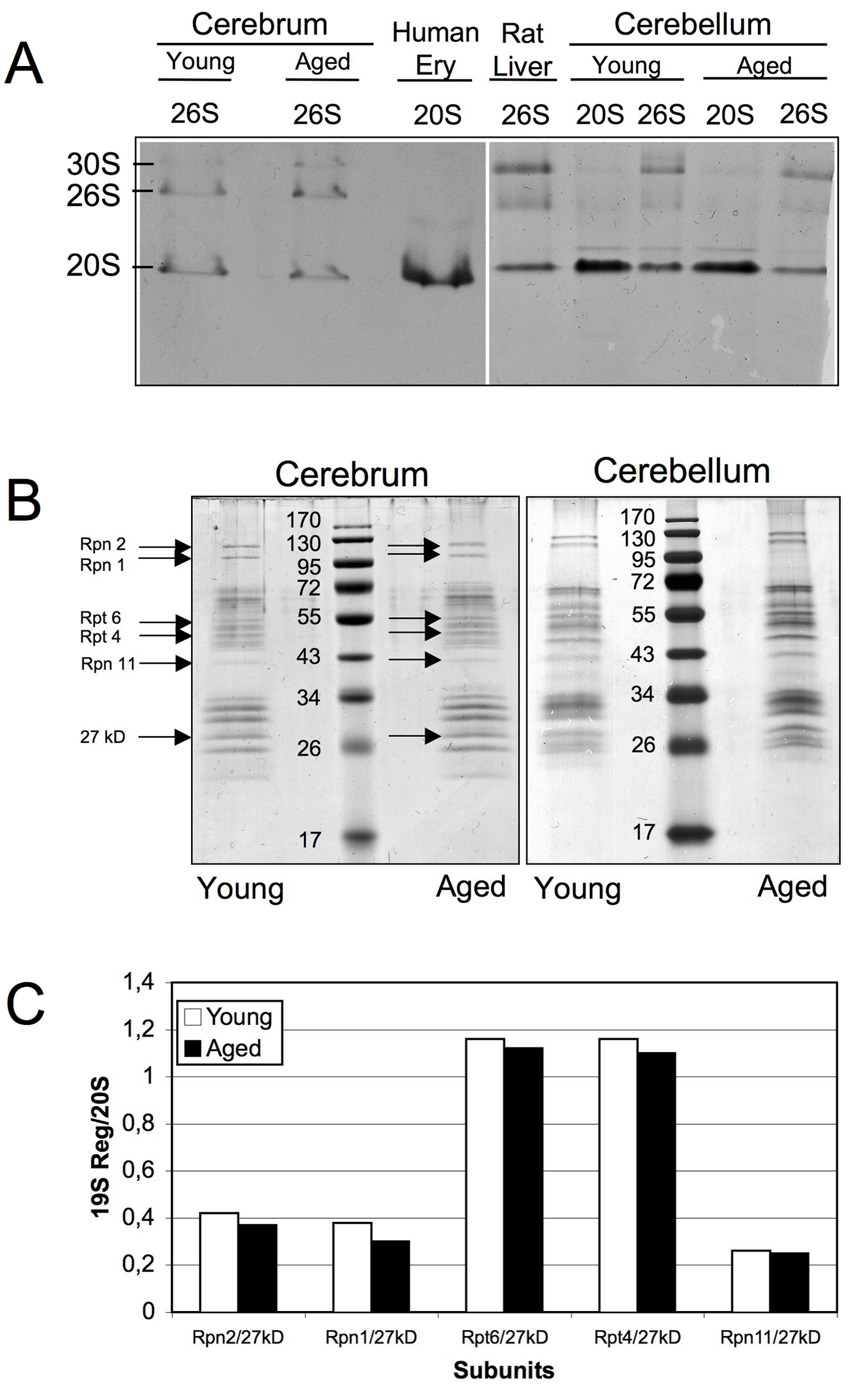

Supplement: Figure S3 — Non-denaturing and SDS-PAGE of 26S proteasomes purified from brain tissues. Panel A. From young and aged rats 4 µg each of 26S proteasome purified from cerebrum and 4 µg each of 26S as well as 20S proteasomes purified from cerebellum were subjected to non-denaturing polyacrylamide gel electrophoresis and the gels stained with Coomassie blue. As references 26S proteasome purified from rat liver (4 µg) and 20S proteasome purified from human erythrocytes (3 µg) were used. Panel B. 2 µg each of 26S proteasome purified from cerebrum and cerebellum of young and aged rats was subjected to SDS polyacrylamide gel electrophoresis and the gels stained with Coomassie blue. Molecular mass standards of 17–170 kD were run in parallel. Panel C. The protein amounts of 19S regulator subunits (indicated according to Shibatani et al 2006) and a 20S proteasome subunit (27 kD) in panel C were densitometrically measured in cerebral 26S proteasomes by the ImageJ software and the ratio of 19S Reg subunits to the 27 kD was calculated. The data show similar ratios for 19S Reg/20S proteasome in 26S proteasomes from young and aged rats. Similar data were obtained for cerebellar 26S proteasomes (data not shown). (TIF) [file pone.0064042.s003.tif]

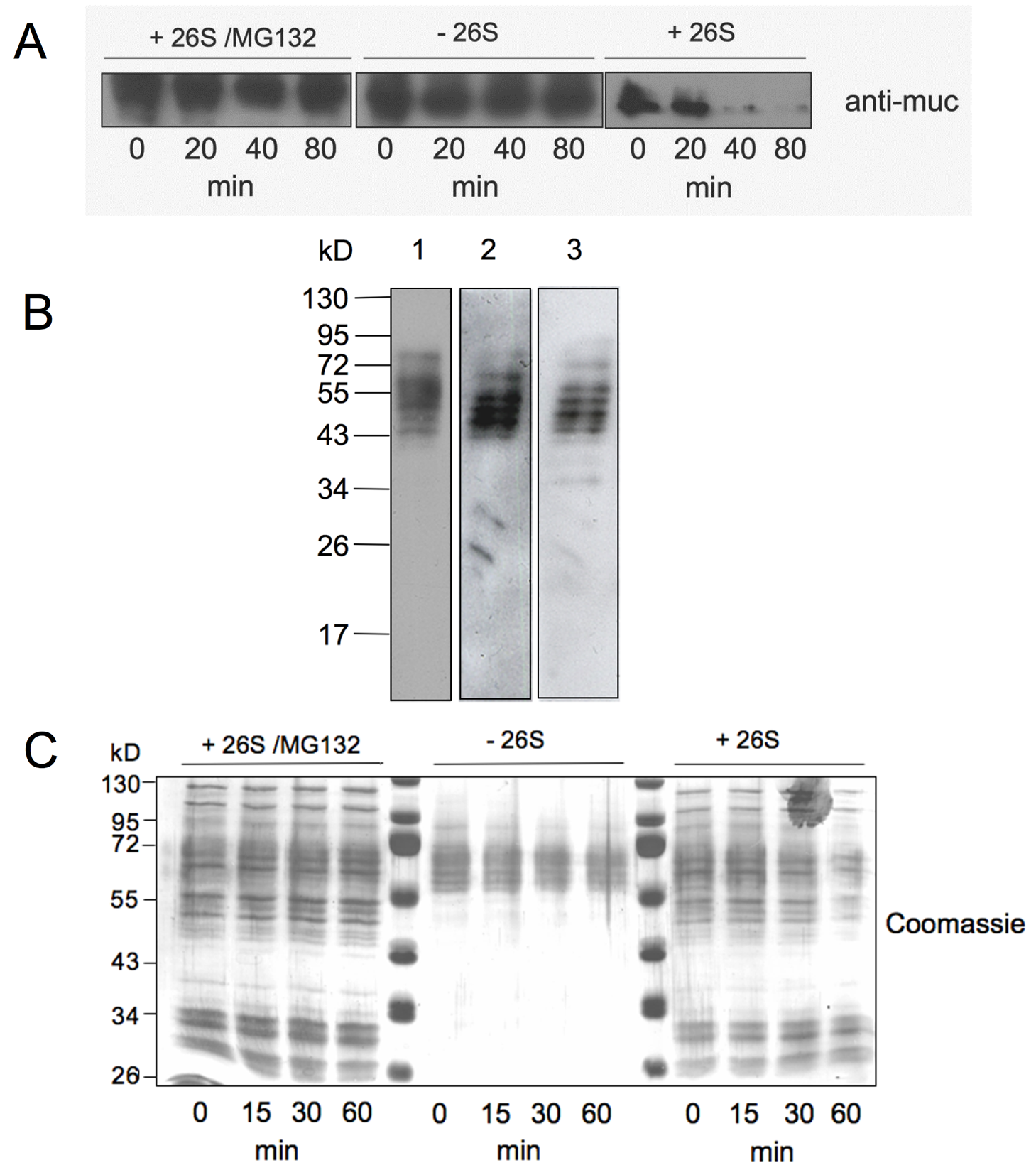

Supplement: Figure S4 — Stability of Ub5Muc4 and polyUb-GST-UbcH5 in the presence and absence of 26S proteasome. Panel A. 600 nM Ub5Muc4 was incubated (at 37°C) with 60 nM 26S proteasome in the presence of 100 µM proteasome inhibitor MG132 (+26S/MG132), without 26S proteasome (−26S) and with 60 nM 26S proteasome (+26S) purified from human erythrocytes. At the times indicated aliquots of the reaction mixture were removed and subjected to SDS-PAGE. Afterwards Ub5Muc4 was detected on immunoblots with an antibody raised against Muc950–958 peptide. Panel B. Poly-Ub-GST-UbcH5 (0.5 µg/lane) was subjected to SDS-PAGE, blotted and then detected by use of antibodies to UbcH5 (lane 1), poly-Ub (lane 2), and GST (lane 3), respectively. Panel C. 800 nM polyUb-GST-UbcH5 was incubated with 60 nM 26S proteasome in the presence of 100 µM proteasome inhibitor MG132 (+26S/MG132), without 26S proteasome (−26S) and with 60 nM mM 26S proteasome (+26S) from human erythrocytes at 37°C. At the times indicated aliquots of the reaction mixture were removed and subjected to SDS-PAGE and remaining poly-Ub-GST-UbcH5 was detected on immunoblots with an antibody raised against UbcH5. (TIF) [file pone.0064042.s004.tif]
